# Prognosis of type 1 autoimmune pancreatitis after corticosteroid therapy-induced remission in terms of relapse and diabetes mellitus

**DOI:** 10.1371/journal.pone.0188549

**Published:** 2017-11-22

**Authors:** Masaki Miyazawa, Hajime Takatori, Tetsuro Shimakami, Kazunori Kawaguchi, Kazuya Kitamura, Kuniaki Arai, Koichiro Matsuda, Taku Sanada, Takeshi Urabe, Katsuhisa Inamura, Takashi Kagaya, Hideki Mizuno, Uichiro Fuchizaki, Taro Yamashita, Yoshio Sakai, Tatsuya Yamashita, Eishiro Mizukoshi, Masao Honda, Shuichi Kaneko

**Affiliations:** 1 Department of gastroenterology, Kanazawa University Hospital, Kanazawa, Japan; 2 Department of gastroenterology, Keiju Medical Center, Nanao, Japan; 3 Department of internal medicine, Toyama Prefectural Central Hospital, Toyama, Japan; 4 Department of internal medicine, Fukui-ken Saiseikai Hospital, Fukui, Japan; 5 Department of gastroenterology, Public Central Hospital of Matto Ishikawa, Hakusan, Japan; 6 Department of internal medicine, Tonami General Hospital, Tonami, Japan; 7 Department of gastroenterology, National Hospital Organization Kanazawa Medical Center, Kanazawa, Japan; 8 Department of gastroenterology, Toyama City Hospital, Toyama, Japan; Baylor College of Medicine, UNITED STATES

## Abstract

**Background and aim:**

Relapse and diabetes mellitus (DM) are major problems for the prognosis of autoimmune pancreatitis (AIP). We examined the prognosis of type 1 AIP after corticosteroid therapy (CST)-induced remission in terms of relapse and DM.

**Methods:**

The study enrolled 82 patients diagnosed with type 1 AIP who achieved remission with CST. We retrospectively evaluated the relapse rate in terms of the administration period of CST, clinical factors associated with relapse, and the temporal change in glucose tolerance.

**Results:**

During follow-up, 32 patients (39.0%) experienced relapse. There was no significant clinical factor that could predict relapse before beginning CST. AIP patients who ceased CST within 2 or 3 years experienced significantly earlier relapse than those who had the continuance of CST (*p* = 0.050 or *p* = 0.020). Of the 37 DM patients, 15 patients (40.5%) had pre-existing DM, 17 (45.9%) showed new-onset DM, and 5 (13.5%) developed CST-induced DM. Patients with new-onset DM were significantly more likely to show improvement (*p* = 0.008) than those with pre-existing DM.

**Conclusions:**

It was difficult to predict relapse of AIP based on clinical parameters before beginning CST. Relapse was likely to occur within 3 years after the beginning of CST and maintenance of CST for at least 3 years reduced the risk of relapse. The early initiation of CST for AIP with impaired glucose tolerance is desirable because pre-existing DM is refractory to CST.

## Introduction

Yoshida *et al*. [1] first introduced autoimmune pancreatitis (AIP) as a new clinical entity of chronic pancreatitis in 1995. Hamano *et al*. [2] identified IgG4 antibody as a useful serum biomarker for diagnosing AIP. Over the last decade, progress in clinicopathological studies has advanced our understanding of AIP. In 2011, a international group developed the International Consensus Diagnostic Criteria (ICDC), which identifies two distinct pathological subtypes with different clinical phenotypes: type 1 and type 2 AIP [3]. Type 1 AIP comprises the great majority of AIP in Asia, including Japan, and tends to be an indication for corticosteroid therapy (CST) compared with type 2 AIP [4].

CST for type 1 AIP consisting of remission induction and maintenance therapy have been established to a degree by building consensuses based on several large-scale studies [4,5]. Hart *et al* provided a treatment strategy with a clear induction regimen, dosage schedule and suggestions for when to start maintenance therapy [4]. However, some questions about the prognosis of type 1 AIP after CST-induced remission remain. Many patients experience relapse either after the tapering or the cessation of CST [4–6], while relapse sometimes occurs during maintenance in some patients. There is no clear consensus on the factors that predict relapse or are associated with the time to relapse. Relatively long-term administration of CST is recommended for preventing relapse, although the appropriate administration period of CST is still unclear. It is not clear whether CST can be stopped after a maintenance period. Furthermore, AIP has a risk of diabetes mellitus (DM) as a complication [7–9]. In this study, we examined the long-term prognosis of type 1 AIP after CST-induced remission, focusing on relapse and DM.

## Materials and methods

### Subjects

This retrospective study was approved by the Ethics Committee of Kanazawa University Hospital. Patients who were diagnosed with type 1 AIP in Kanazawa University Hospital or its affiliated institutes from January 2000 to May 2015 and were followed for more than 1 year after CST-induced remission were enrolled. The diagnosis of type 1 AIP was based on the ICDC.^3^ This study included patients who met the criteria for definitive or probable AIP. The medical charts were reviewed and clinical data were collected to evaluate various factors associated with the prognosis of type 1 AIP. All patient data were completely anonymized, deidentified, and aggregated before analysis.

### Definitions

In this study, CST-induced remission was defined as the improvement of clinical symptoms in addition to the resolution of pancreatic parenchymal enlargement or other organ involvement (OOI) on cross-sectional radiological imaging within 3 months after the initiation of CST.^10^ A wide variety of CST regimens were used for maintenance therapy based on each patient’s condition. In this study, maintenance therapy was defined as continuous administration for more than 6 months after the initiation of CST. The maintenance dose was defined as the daily oral prednisolone dose administered for the longest term during the follow-up period, ranging from 5 mg/day to 10 mg/day. Patients underwent cross-sectional radiological imaging at least once every six months basically. When clinical symptoms such as abdominal pain, back pain and jaundice were observed, extraordinary imaging was performed as needed. Serum IgG and IgG4 level were measured once every three to six months. Relapse was defined as the recurrence of clinical symptoms in addition to the exacerbation of pancreatic parenchymal enlargement or OOI on cross-sectional radiological imaging after CST-induced remission [10]. In this study, the re-elevation of serum IgG or IgG4 level only was not regarded as relapse and oral prednisolone dose was not increased in such patients. We excluded patients who experienced relapse during tapering CST within 3 months from this study because we focused on evaluating the necessity of maintenance therapy for AIP.

In this study, DM was diagnosed by the diagnostic criteria of the Japan Diabetes Society [11]. We classified patients with DM by the style of onset: pre-existing DM, new-onset DM and CST-induced DM. Pre-existing DM was defined as DM for which diet therapy or medication was started more than 1 year before the diagnosis of AIP and diagnosed by past records of laboratory tests such as fasting glucose level, casual glucose level and/or the HbA1c level. New-onset DM was defined as DM that occurred simultaneously with the diagnosis of AIP or DM with indeterminate onset time. CST-induced DM was defined as diagnosed and treated after the initiation of CST. These two types of DM were diagnosed by newly-checked laboratory tests such as fasting glucose level, casual glucose level and/or the HbA1c level. The HbA1c level was calculated as the value of the National Glycohemoglobin Standardization Program, which is equivalent to the value of the Japan Diabetes Society + 0.4% [11]. We defined insulin secretory dysfunction as homeostasis model assessment (HOMA) for beta cell function of less than 20 or 24-hour urinary C-peptide of less than 20 μg/day, and insulin resistance as HOMA for insulin resistance of more than 2.5 or fasting blood insulin level of more than 15 μU/ml.

### Statistical analysis

Categorical variables were compared using the chi-square test. Continuous variables were analyzed using the Student’s *t*-test or Mann–Whitney *U*-test. Survival probabilities were calculated using the Kaplan–Meier method with the log-rank test to evaluate the period to relapse of AIP. Differences with *p*-values of less than 0.05 were considered to be statistically significant. The statistical analyses were performed using StatView (SAS Institute, Cary, NC, USA).

## Results

### Characteristics of the type 1 AIP patients

The study included 82 patients with type 1 AIP. Table 1 summarizes the clinical characteristics of the patients. Based on the ICDC, 79 patients (96.3%) were judged to have definitive type 1 AIP, 3 (3.7%) had probable type 1 AIP. Of the 17 patients whose histology of the pancreas was obtained, 5 patients had level 1 lymphoplasmacytic sclerosing pancreatitis (LPSP) and 12 patients had level 2 LPSP. Diagnostic steroid trial was done in 10 patients.

**Table 1:** Clinical characteristics of the patients with type 1 autoimmune pancreatitis.

Parameter	
Age, mean±SD, years	65.6±11.0
Sex, male / female (%)	73 (89.0) / 9 (11.0)
Body mass index, mean±SD, kg/m^2^	22.1±2.7
Obstructive jaundice^†^, present / absent (%)	23 (28.0) / 59 (72.0)
Serum IgG levels, median (range), mg/dl	1839 (770–4941)
Serum IgG levels of 1,800 mg/dl or more (% of 75 patients)	40 (53.3)
Serum IgG4 levels, median (range), mg/dl	381.5 (117–4280)
Serum IgG4 levels of 550 mg/dl or more (% of 78 patients)	26 (33.3)
Pancreatic parenchymal enlargement, diffuse / segmental (%)	54 (65.9) / 28 (34.1)
Other organ involvement^‡^, present / absent (%)	35 (42.7) / 47 (57.3)
Initial prednisolone doses, median (range), mg/day	30 (20–40)
Maintenance therapy, present / absent (%)	76 (92.7) / 6 (7.3)
Maintenance prednisolone doses, median (range), mg/day	5 (2.5–10)
Relapse, present / absent (%)	32 (39.0) / 50 (61.0)
Follow-up period^§^, median (range), months	52.9 (13.1–180.4)

^†^ Serum total bilirubin levels of more than 3.0 mg/dl with dilation of bile duct.

^‡^ Biliary strictures located proximal to the intrapancreatic portion of the common bile duct, retroperitoneal fibrosis, sialadenitis or and renal involvement in type 1 AIP.

^§^ Follow-up period counts from the initiation of CST.

CST, corticosteroid therapy; DM, diabetes mellitus

### Corticosteroid treatment for type 1 AIP

The median follow-up period was 52.9 (range 13.1–180.4) months. The initiation of CST was started at 40 mg/day in 26 patients (31.7%), 35 mg/day in 3 patients (3.7%), 30 mg/day in 46 patients (56.1%), 20 mg/day in 7 patients (8.5%). The prednisolone dose was tapered gradually, by 2.5–10 mg every 1–8 weeks, until the dose reached the maintenance dose or zero in principle. In this study, all patients achieved remission while tapering CST. 76 of the 82 patients (92.7%) had maintenance therapy and 6 (7.3%) had not. Whether maintenance of CST is continued or stopped in AIP patients was decided by individual doctor’s judgement based on side effect, background disease or patient’s request. The median duration of CST was 31.8 (range 1.4–179.3) months. 68 (82.9%) of the 82 patients who were followed up had CST for more than 1 year, 31 (66.0%) of the 47 for more than 3 years, and 11 (55.0%) of the 20 for more than 5 years.

The median maintenance prednisolone dose was 5 (range 2.5–10) mg/day. Of the 76 patients who had maintenance therapy, 54 (71.1%) had a maintenance dose of 5 mg/day or more and 22 (28.9%) had a dose of 2.5 to 5 mg/day. CST was stopped during follow-up in 36 patients (43.9%).

### Relapse

During the follow-up period, relapse occurred in 32 of the 82 type 1 AIP patients (39.0%) after CST-induced remission. Table 2 shows the clinical characteristics of the patients with or without relapse evaluated by univariate analyses. There was no significant clinical factor that could predict of relapse before beginning CST. Patients who were male (*p* = 0.273) and young (*p* = 0.211) relatively tend to experience relapse. Neither the serum IgG levels (*p* = 0.513) nor IgG4 levels (*p* = 0.500) predicted relapse.

**Table 2:** Clinical characteristics of the patients with or without relapse.

Parameter	Relapse (+)	Relapse (-)	*p* value
Age, mean±SD, years	63.7±12.0	66.8±10.3	0.211
Sex, male / female	30 / 2	43 / 7	0.273
Body mass index, mean±SD, kg/m^2^	22.0±2.8	22.2±2.7	0.740
Obstructive jaundice^†^, present / absent	9 / 23	14 / 36	0.990
Serum IgG levels, median (range), mg/dl	1898 (770–4941)	1808 (1115–3727)	0.513
Serum IgG4 levels, median (range), mg/dl	397 (117–4280)	379.5 (140–2140)	0.500
Serum IgG4/IgG ratio	0.26 (0.07–0.94)	0.22 (0.07–0.52)	0.352
Pancreatic parenchymal enlargement, diffuse / segmental	22 / 10	32 / 18	0.658
Narrowing of main pancreatic duct on ERP, diffuse / segmental	19 / 8	27 / 8	0.546
Other organ involvement^‡^, present / absent	12 / 20	23 / 27	0.448
Biliary strictures located proximal to the intrapancreatic portion, present / absent	4 / 28	7 / 43	0.846
Retroperitoneal fibrosis, present / absent	3 / 29	4 / 46	0.828
Sialadenitis, present / absent	10 / 22	15 / 35	0.905
Initial prednisolone doses, median (range), mg/day	30 (20–40)	30 (20–40)	0.850
Daily doses per body weight, median (range), mg/kg	0.53 (0.28–0.74)	0.54 (0.33–0.78)	0.712
Maintenance therapy, present / absent	30 / 2	46 / 4	0.767
Maintenance prednisolone doses of 5mg/day or more, present / absent	19 / 11	35 / 11	0.231
Continuance of corticosteroid therapy during follow-up period^§^	16 /16	30 / 20	0.373

^†^ Serum total bilirubin levels of more than 3.0 mg/dl with dilation of bile duct.

^‡^ Biliary strictures located proximal to the intrapancreatic portion of the common bile duct, retroperitoneal fibrosis, sialadenitis or and renal involvement in type 1 AIP.

^§^ Follow-up period counts from the initiation of corticosteroid therapy.

ERP, endoscopic retrograde pancreatography

Maintenance prednisolone doses of 5 mg/day or more (*p* = 0.231) and the continuance of CST during follow-up period (*p* = 0.373) had a lower incidence of relapse, although this difference was not significant.

**Fig 1:**
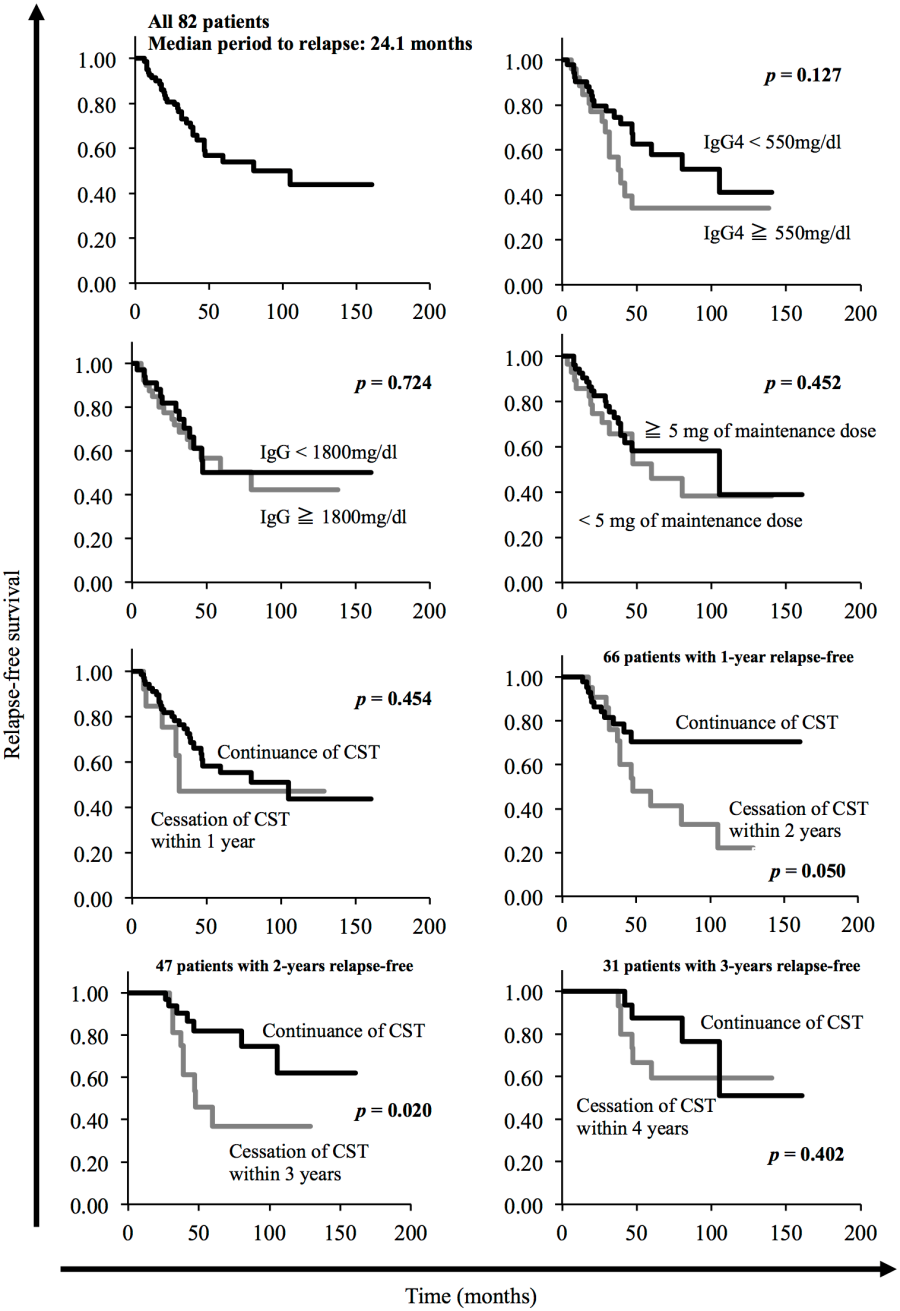
The period from initiation of corticosteroid therapy to relapse of AIP. Kaplan–Meier plots of the cumulative relapse rate of type 1 autoimmune pancreatitis grouped for various parameters. Patients who had an elevated serum IgG4 level of 550 mg/dL or more (*p* = 0.127) tended to experience early relapse. Serum IgG level of 1800 mg/dLor more (*p* = 0.724) and maintenance dose of 5 mg/day or more (*p* = 0.452) were not associated with early relapse. Grouped for administration period of CST, AIP patients who ceased CST within 2 or 3 years experienced significantly earlier relapse than those who had the continuance of CST (*p* = 0.050 or *p* = 0.020).

The median period from the initiation of CST to relapse was 24.1 (range 3.2–105.2) months. Of the 32 patients who relapsed, 8 (25.0%) relapsed within 1 year, 8 (25.0%) between 1 to 2 years, 6 (18.8%) between 2 to 3 years, 7 (21.9%) between 3–4 years, and 3 (9.4%) after 4 years. The longest period from the initiation of CST to relapse was 105.2 months, and this patient relapsed after stopping CST after about 8 years of maintenance therapy. Fig 1 shows Kaplan–Meier plots of the cumulative relapse rate grouped for various parameters. We determined the optimal cut-off values for the serum IgG4 and IgG levels using receiver operating characteristic (ROC) curves. Patients who had an elevated serum IgG4 level of 550 mg/dL or more (*p* = 0.127) tended to experience early relapse, although there was no significant association. Serum IgG level of 1800 mg/dLor more (*p* = 0.724) and maintenance dose of 5 mg/day or more (*p* = 0.452) were not associated with early relapse. Grouped for the administration period of CST, there was no significant difference between the period from the initiation of CST to relapse in patients who continued CST and ceased it within 1 year (*p* = 0.454). However, AIP patients who ceased CST within 2 or 3 years experienced significantly earlier relapse than those who had the continuance of CST (*p* = 0.050 or *p* = 0.020). The cessation of CST after 3 years from the initiation n of CST was not a significant factor associated with the time to relapse. In 16 patients with relapse after cessation of CST, 10 and 6 received CST for more than 1and 2 years, respectively. There was no significant difference between the duration of CST and the period from cessation of CST to relapse (more than 1 year or not; *p* = 0.386, more than 2 years or not; *p* = 0.588).

### Correlation between DM and type 1 AIP with CST-induced remission

We evaluated the change in glucose tolerance over time in 60 patients. During follow-up, 37 patients (61.7%) had DM. Of the 37 DM patients, 15 patients (40.5%) had pre-existing DM, 17 (45.9%) showed new-onset DM, and 5 (13.5%) developed CST-induced DM. Table 3 shows the associations between the presence of DM and the clinical characteristics of the type 1 AIP patients. There was no significant clinical and treatment factor associated with the presence of DM.

**Table 3:** The association between the presence of diabetes mellitus and the clinical characteristics of the type 1 autoimmune pancreatitis.

Parameter	All (n = 60)	Style of onset			Non-DM (n = 23)	*p* value DM vs Non-DM
		Pre-existing (n = 15)	New-onset (n = 17)	CST-induced (n = 5)		
Age, mean±SD, years	65.1±10.9	66.7±9.4	65.6±10.9	65.4±17.1	63.6±10.9	0.38
Sex, male / female	54 / 6	14 / 1	13 / 4	5 / 0	22 / 1	0.25
Body mass index, mean±SD, kg/m^2^	21.9±2.5	21.8±3.2	21.9±1.9	23.8±4.4	21.4±1.6	0.24
Serum IgG levels, median (range), mg/dl	1878 (1115–4941)	1795 (1181–4547)	1792.5 (1115–2860)	2088 (1712–2720)	2025 (1265–4941)	0.19
Serum IgG4 levels, median (range), mg/dl	378 (140–4280)	395.5 (155–4280)	358 (140–1170)	752 (483–1040)	354 (198–1610)	0.69
Enlargement of the pancreas, diffuse / segmental	42 / 18	11 / 4	13 / 4	3 / 2	15 / 8	0.52
Initial prednisolone doses, median (range), mg/day	30 (20–40)	30 (30–40)	30 (20–40)	30 (30–40)	30 (20–40)	0.24

DM, diabetes mellitus; CST, corticosteroid therapy

We evaluated the clinical course of DM based on the change in the HbA1c level or diabetic medications at the time that the prednisolone dose was decreased to maintenance dose or less and classified it as improvement, no change or exacerbation. Improvement was defined as a decrease in the HbA1c level by more than 0.5% without strengthening the therapy for DM (*i*.*e*., starting diabetes medication, increasing the insulin injection dose, adding new oral hypoglycemic agents, or switching from oral hypoglycemic agents to insulin injection) compared with the condition before the initiation n of CST. Exacerbation was defined as an increase in the HbA1c level by more than 0.5% or no decrease despite strengthening the therapy for DM compared with the condition before the initiation of CST. The other clinical course was judged as no change. Table 4 shows the clinical course of DM grouped for the type of onset of DM.

**Table 4:** The clinical course of diabetes mellitus grouped for the type of onset.

Style of onset	Clinical course of DM			*p* value Improvement vs Others	Medication for DM		*p* value
	Improvement	Exacerbation	No change		Continuance	Cessation	
Pre-existing (n = 15)	2 (13.3%)	4 (26.7%)	9 (60.0%)	0.008*	15 (100%)	0 (0%)	0.01*
New-onset (n = 17)	10 (58.8%)	3 (17.6%)	4 (23.5%)		11 (64.7%)	6 (35.3%)	
CST-induced (n = 5)	-	5 (100%)	-	-	4 (80%)	1 (20%)	-

We classified the clinical course of DM as improvement, no change or exacerbation.

Improvement was defined as a decrease in the HbA1c level by more than 0.5% without strengthening the therapy for DM (i.e., starting diabetes medication, increasing the insulin injection dose, adding new oral hypoglycemic agents, or switching from oral hypoglycemic agents to insulin injection) compared with the condition before the initiation of CST. Exacerbation was defined as an increase in the HbA1c level by more than 0.5% or no decrease despite strengthening the therapy for DM compared with the condition before the initiation of CST. The other clinical course was judged as no change.

DM, diabetes mellitus; CST, corticosteroid therapy.

* p < 0.05

First, 5 patients with CST-induced DM were classified as exacerbation. All of them showed borderline HbA1c level (5.6–6.4%) before the initiation of CST. Of the 32 patients excluding the group with CST-induced DM, 12 (37.5%) showed improvement, 7 (21.9%) showed exacerbation, and 13 (40.6%) showed no change. Patients with new-onset DM were significantly more likely to show improvement (*p* = 0.008), and achieve the medication-free (*p* = 0.01) status than those with pre-existing DM.

We evaluated the presence of insulin secretory dysfunction and insulin resistance before the initiation of CST in 13 patients with new-onset DM, but could not in patients with pre-existing DM because of the influence of diabetic medication. 4 of 13 patients with new-onset DM showed insulin secretory dysfunction, while the others showed neither of them. 3 of 4 patients with insulin secretory dysfunction showed improvement in the clinical course of DM after the maintenance of CST.

We also followed the change in HbA1c over time in 23 non-diabetic patients. 16 patients (69.6%) showed borderline HbA1c (5.6–6.4%) before the initiation of CST and only one patient showed exacerbation in glucose tolerance.

## Discussion

Since the Japanese diagnostic criteria for AIP were established in 2002 [12], CST has been administered in many patients. Although CST-induced remission is achieved in about 98% of AIP patients, many patients will experience relapse [5]. Hart *et al*. reported that relapse was more common in type 1 AIP (35.8%) than in type 2 AIP (15.3%) [4].

The importance of maintenance therapy for type 1 AIP is well known in terms of the prevention of relapse. A Japanese multicenter randomized controlled trial of long-term CST showed that the relapse rate of patients who continued maintenance therapy for 3 years was 23.3% versus 57.9% for patients who stopped it at 26 weeks [13]. However, there is a great difference in the use of maintenance therapy between Japan and Western countries. The Mayo Clinic adopted a protocol with completion of CST in 11 weeks and reported that the relapse rate of AIP with sclerosing cholangitis was 53% [14]. Raina *et al*. reported that the relapse rate of AIP with CST for only 12 weeks was 60% [15]. In our study, the presence of maintenance therapy for more than 6 months did not reduce total relapse rate significantly because the cumulative relapse rate of AIP would increase if the follow-up period become longer. We should consider the period of follow-up and administration of CST. Our results obtained by the long-term observation with a median of 52.9 months showed the group with the cessation of CST within 2 or 3 years had significantly higher cumulative relapse rate than that with the continuance of CST. This result was not very different from other reports [4–6,16–20].

A Japanese research group recommended continuing CST possibly up to 3 years in terms of the prevention of relapse since a previous study revealed that 92% of the relapses occurred within 3 years [5,21]. However, our results that nearly 30% of the patients experienced relapse after follow-up for 3 years was slightly different from the results of previous studies [5,20]. Some patients, especially those who stopped maintenance therapy, may experience relapse after long-term follow-up. We should be aware that relapse of AIP might occur even if time past from cessation of CST.

If we can predict whether AIP will relapse before treatment, it would help us to determine the administration period of CST. Previous studies reported some clinical factors that might predict relapse, including the presence of IgG4-related cholangitis [4,22,23], high serum IgG4 levels [14], the IgG levels [24], diffuse pancreatic parenchymal enlargement [25,26], diffuse narrowing of the main pancreatic duct [27], duodenal papillitis [16], and OOI [27]. In our study, these clinical factors were not the independent predictor of relapse. It seems difficult to identify patients who will experience relapse of AIP using a single clinical value before the initiation of CST.

Since AIP can progress to irreversible pancreatic insufficiency [9], we recommend adequate maintenance therapy for type 1 AIP with pancreatic complications. The maintenance of CST for 3 years seems standard. Having said that, CST for further longer period is desirable because relapse may occur after long time passes. To investigate appropriate administration period of CST, a prospective randomized controlled trial with observation for quite a long time should be conducted on condition that the way of maintenance of CST is decided by defined criteria. If a system for scoring the disease activity based on multiple parameters with high sensitivity and specificity for predicting relapse were established, it would be possible to decide the appropriate way of CST individually.

Furthermore, we must be aware that both AIP itself and CST have a risk of DM. According to previous reports, 40–80% of AIP patients have DM [7–9,18,28–30]. Type 1 AIP has DM more frequently than type 2 AIP [31]. In our study, 61.7% of the type 1 AIP patients had During the follow-up. A national survey by Nishimori *et al*. [7] reported that 55% of the patients with new-onset DM showed improvement and 16% showed exacerbation after the initiation of CST; 36% of the patients with pre-existing DM showed improvement and 18% showed exacerbation. Miyamoto *et al*. [8] stated that a long-term positive effect of CST on glucose tolerance might be greater than a short-term negative effect countering the effect of insulin. The early initiation of CST for AIP with impaired glucose tolerance before the development of irreversible endocrine dysfunction is desirable because pre-existing DM concurrent with AIP is refractory to CST. As our study showed, new-onset DM was likely to be more responsive to CST than pre-existing DM. CST for AIP with new-onset DM can eventually achieve a medication-free status. Anti-inflammatory effect of CST may improve insulin secretory dysfunction associated with AIP, but on the other hand we must remember some AIP patients develop DM after the initiation of CST. It is notable that all of patients with CST-induced DM in our study showed borderline HbA1c level before treatment. Even then it is difficult to predict the exacerbation of DM caused by CST beforehand. In patients with uncontrollable exacerbation of DM, cessation of CST after clinical remission will be permissible. Instead, these patients should be observed paying attention to relapse of AIP.

There are some limitations to this study. First, our study design was not prospective. Second, different diagnostic criteria for AIP are used in different studies. We adopted the ICDC, whereas other studies have used the Asian criteria [8,9,16,20,32]. Moreover, there is no systematic standardized protocol for CST among institutions. Whether maintenance of CST is continued or stopped in AIP patients was not decided by defined criteria. Regarding DM, we should consider the difference of the way to evaluate glucose tolerance from previous studies. HbA1c level is easy to measure in outpatient clinic and useful for evaluation of long-term glucose tolerance, but not enough in that it may be affected by individual differences or background diseases and cannot reflect the circadian change of blood glucose level.

In conclusion, we examined recent information about the prognosis of type 1 AIP focusing on CST and DM as a complication. Since some problems regarding about relapse and DM remain, further investigation is required.
